# Supplementary material for: Transcriptome Analysis of Genes Involved in Cold Hardiness of Peach Tree (Prunus persica) Shoots during Cold Acclimation and Deacclimation
Source: Genes (Basel). 2020 Jun 1;11(6):611. doi: 10.3390/genes11060611 (PMC7349757; doi:10.3390/genes11060611)
Supplement: Supplementary file 1 [file genes-11-00611-s001.zip › Figure S1-S3.docx]

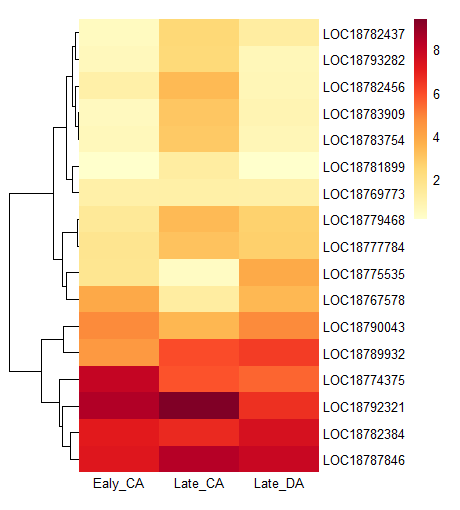


**Figure S1.** Heatmap of the log_2_(FPKM + 1) values of six *xyloglucan endotransglucosylase/hydroxylase* (*XEH*) transcripts [*XEH2*s (LOC18781899, LOC18782437, LOC18782456, LOC18783754, LOC18783909, and LOC18787846)], two *XEH protein* transcripts [*XEH9* (LOC18782384) and *XEH31* (LOC18775535)], and nine *probable XEH* (*pXEH*) *protein* transcripts [*pXEHB* (LOC18790043), *pXEH6* (LOC18774375), *pXEH8* (LOC18779468), *pXEH10* (LOC18767578), *pXEH23*s (LOC18792321 and LOC18793282), *pXEH26* (LOC18769773), *pXEH28* (LOC18789932), and *pXEH30* (LOC18777784)] during cold acclimation (CA), from early CA to late CA, and deacclimation (DA), from late CA to late DA, in the ‘Soomee’ peach tree shoots. The differentially expressed genes were clustered based on their FPKM values.


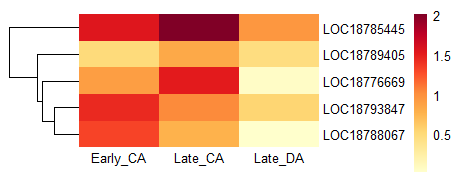


**Figure S2.** Heatmap of the log_2_(FPKM + 1) values of five *dehydration-responsive element-binding protein* (*DREB*) transcripts [*DREB1A* (LOC18778067), *DREB1E* (LOC18776669), *DREB2C* (LOC18785445), *DREB2D* (LOC18789405), and *DREB3* (LOC18793847)] during cold acclimation (CA), from early CA to late CA, and deacclimation (DA), from late CA to late DA, in the ‘Soomee’ peach tree shoots. The differentially expressed genes were clustered based on their FPKM values.

**Figure S3.** Changes in the log_2_(FPKM +1) values in *low-temperature-induced 65 kDa protein* (LOC18786412), *late embryogenesis abundant protein D-29* (LOC18788434), *dehydrin Xero 2* (LOC18769991), and *desiccation-related protein PCC13-62* (LOC18789656) transcripts during cold acclimation (CA), from early CA to late CA, and deacclimation (DA), from late CA to late DA, in the ‘Soomee’ peach tree shoots. Vertical bars are the standard errors of the means (*n* = 3). Different letters indicate significant differences among the three physiological stages within the same DEGs using the Duncan’s multiple range test at *P* < 0.05.
